# Unresolved intramuscular inflammation, not diminished skeletal muscle regenerative capacity, is at the root of rheumatoid cachexia: insights from a rat CIA model

**DOI:** 10.14814/phy2.15119

**Published:** 2021-11-21

**Authors:** Tracey Ollewagen, Yigael S. L. Powrie, Kathryn H. Myburgh, Carine Smith

**Affiliations:** ^1^ Department Physiological Sciences Science Faculty Stellenbosch University Stellenbosch South Africa; ^2^ Division of Clinical Pharmacology Department of Medicine Faculty of Medicine and Health Sciences Stellenbosch University Stellenbosch South Africa

**Keywords:** cachexia, collagen‐induced arthritis, macrophage, myofibroblast, satellite cell

## Abstract

Rheumatoid arthritis targets numerous organs in patients, including the skeletal muscle, resulting in rheumatoid cachexia. In the muscle niche, satellite cells, macrophages, and myofibroblasts may be affected and the factors they release altered. This study aimed to assess these cell types, cytokines, and growth factors and their relationships to muscle fiber size and number in a rodent collagen‐induced arthritis (CIA) model, in order to identify new therapeutic targets. Fiber cross‐sectional area (CSA) was 57% lower in CIA than controls (*p *< 0.0001), thus smaller but more fibers visible per field of view. Immunostaining indicated the increased presence of satellite cells, macrophages, myofibroblasts, and myonuclei per field of view in CIA (*p *< 0.01), but this finding was not maintained when taking fiber number into consideration. Western blots of *gastrocnemius* samples indicated that tumor necrosis factor‐α was significantly elevated (*p *< 0.01) while interleukin‐10 (IL‐10) was decreased (*p *< 0.05) in CIA. This effect was maintained (and heightened for IL‐10) when expressed per fiber number. Myogenic regulatory factors (MyoD and myogenin), transforming growth factor‐β and inhibitor of differentiation were significantly elevated in CIA muscle and levels correlated significantly with CSA. Several of these factors remained elevated, but bone morphogenetic protein‐7 decreased when considering fiber number per area. In conclusion, CIA‐muscle demonstrated a good regenerative response. Myoblast numbers per fiber were not elevated, suggesting their activity results from the persistent inflammatory signaling which also significantly hampered maintenance of muscle fiber size. A clearer picture of signaling events at cellular level in arthritis muscle may be derived from expressing data per fiber.

## Graphical abstract

Collagen‐induced arthritis in rodents simulate rheumatoid cachexia in humans. Skeletal muscle fibers exhibit decreased size, but not decreased number. Data illustrates that the unresolved inflammatory cellular environment hampers muscle maintenance, but that muscle fibers still have regenerative capacity.

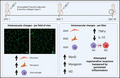

**Abbreviations**
BCA: bicinchroninic acid
BMP‐7: bone morphogenetic protein
CIA: collagen‐induced arthritis
CSA: cross‐sectional area
HRP: horse‐radish peroxidase
Id2: inhibitor of differentiation
IL: interleukin
Mafbx: muscle atrophy f‐box
MCP‐1: monocyte chemoattractant protein
MIF: macrophage migration inhibitory factor
NC: non‐arthritic control
NF‐κB: Nuclear Factor kappa‐light‐chain‐enhancer of activated B cells.
PCNA: proliferating cell nuclear antigen
RA: rheumatoid arthritis
TBS‐T: tris‐buffered saline +tween
TGFβ: transforming growth factor‐β
TNF‐α: tumor necrosis factor‐α

## 1 INTRODUCTION

Rheumatoid arthritis (RA), an inflammatory auto‐immune disease, is not limited to the synovial lining of affected joints. Despite the etiology of RA still requiring elucidation, the disease trigger(s) is known to stimulate multiple cell types, including monocytes, fibroblasts, and T‐cells, as well as the cytokines secreted by these cells, to induce and maintain the immune reaction responsible for the development of RA (Gaffo et al., 2006). The chronic inflammation characterizing this condition affects various organs and tissues, including skeletal muscle, resulting in substantial morbidity and increased risk of premature mortality (Koch, 2007). Addressing these secondary symptoms, such as rheumatoid cachexia (Masuko, 2014; Walsmith & Roubenoff, 2002), may increase the standard of living of these patients significantly. However, the limitations of clinical routine invasive assessments of RA patients are obstacle in elucidating the full pathology at tissue level, warranting investigation using rodent models.

In terms of proinflammatory signaling, tumor necrosis factor (TNF)‐α is one of the key cytokines implicated in the development of RA – its presence in RA joint tissue (Chu et al., 1991) and its potential to degrade cartilage is well‐established (Araki & Mimura, 2016; Dayer et al., 1985). Overall, proinflammatory cytokines are elevated in both plasma and synovial fluid of RA patients. However, the synovial fluid exhibits a greater accumulation of cytokines than blood plasma (Wright et al., 2012). Similarly, plasma cytokine profile also differs from that reported from muscle biopsies of RA patients (Huffman et al., 2017). Together, this suggests that the blood cytokine profile may only represent the clearance of excess cytokines secreted, rather than the actual picture at tissue level. Although there is a paucity of information, muscle inflammatory markers have been associated with disease activity, disability, and pain (Huffman et al., 2017), highlighting their importance in disease progression and the necessity to understand cytokine signaling at the tissue level.

One of the proposed mechanisms of rheumatoid cachexia itself is the lingering proinflammatory state observed in RA (Walsmith & Roubenoff, 2002). For example, in rodent collagen‐induced arthritis (CIA) models, blocking TNF‐α and/or IL‐1β reduced the extent of muscle wasting (Dayer, 2002; Roubenoff et al., 1997), while heightened proinflammatory and reduced anti‐inflammatory cytokines were reported to interfere with regulated satellite cell activation and differentiation, resulting in reduced regenerative capacity (Ollewagen et al., 2021; Teixeira & ON, Filippin LI, Xavier RM, 2012). Furthermore, chronic inflammation also dysregulates the proteins of the ubiquitin‐proteasome pathway, further contributing to muscle wasting (Bodine & Baehr, 2014; Gómez‐SanMiguel et al., 2016), related to the unresolved inflammation, Fibrosis is a common characteristic of RA and fibroblasts are fairly abundant in all tissues (Wynn, 2019), but specifically also skeletal muscle (Agrawal et al., 2003), with a significant amount of fibrosis detected in rodent CIA skeletal muscle (Oyenihi et al., 2019). However, very few investigations consider the potential role of fibroblast signaling to dysregulated muscle mass maintenance in RA, and almost no data are available from a realistic simulation of the human *in vivo* scenario (Ollewagen et al., 2021).

Lastly, the severity and rate of progression of RA and rheumatoid cachexia, as well as individual responses to treatment, are extremely variable (Santo et al., 2018). This complexity of the topic may, at least in part, explain the relative lack of data regarding the specific intramuscular changes, especially from human tissue samples. The use of rodent models of collagen‐induced arthritis to elucidate the intramuscular profile of major cellular role players and cytokine signaling in arthritis, may therefore contribute significantly to our understanding of the extent of dysregulation of intramuscular inflammation and muscle maintenance.

In our opinion, collagen‐induced arthritis (CIA) model is more physiologically relevant for this purpose than the complete Freund's adjuvant model, where inflammation is triggered in part by a bacterial insult, since bacterial infection is not accepted as a major general trigger for RA (although bacterial infection is implicated as a role player in RA associated with periodontal disease specifically (Perricone et al., 2019). While the use of Freund's adjuvant injected into the knee or ankle of mice successfully induced skeletal muscle pathology similar to that of RA patients (Steinz et al., 2019), the type II collagen in complete Freund's adjuvant is the component responsible for triggering T cells, B cells, and production of autoantibodies, simulating auto‐immune disease mechanisms very similar to those observed in human RA development (Asquith et al., 2009; Kannan et al., 2005). Although the model has its limitations, for example, in terms of the time frame for disease and cachexia development, which is much faster than that of human RA, we have previously reported that the rodent CIA‐model realistically mimicked the profile of rheumatoid cachexia in rats (Oyenihi et al., 2019), justifying its use in the current context.

Although studies have focussed on different cell types/responses in isolation through cell culture models, literature still lacks a more comprehensively constructed picture in RA skeletal muscle. To our knowledge, there are no data to provide information on potential changes in relative distribution of different cellular role players in the skeletal muscle of RA patients, specifically those contributing to or affected by rheumatoid cachexia. Myoblasts, fibroblasts, and macrophages interact with one another through the proteins (such as cytokines as growth factors) that they release (Ollewagen et al., 2021) to maintain healthy skeletal muscle. Dysregulation of this process could clearly be detrimental in a RA or chronic inflammatory environment. Therefore, this study aimed to quantify the relative distribution of different relevant cell types in skeletal muscle from a rodent CIA model, in parallel with intramuscularly secreted molecular messengers and indicators of arthritis‐related cachexia.

## 2 MATERIALS AND METHODS

### 2.1 Ethics statement and animal handling

Following approval from the Stellenbosch University Animal Research Ethics Committee (Protocol number: SU‐ACUD17‐00034), twenty (Haddad et al., 2005) female Sprague–Dawley rats weighing 180 – 200 g were obtained from the Stellenbosch University small laboratory animal breeding facility. The choice of using female rats only is based on proof that female rodents are more susceptible to developing RA than males (Song et al., 2015). Rats were housed in groups of five rats per cage in a temperature‐ and humidity‐controlled room (23 ± 1*°*C, 40–60% humidity) with a set 12 h light–dark cycle (lights on at 6am) and fed standard commercially available rat chow and tap water *ad libitum*. After acclimatization, rats were randomly divided into two groups of 10 rats each—non‐arthritis control (NC) and collagen‐induced arthritis (CIA). All experimental animals received humane care according to the principles outlined in the National Research Foundation Guide for Care and Use of Laboratory Animals.

### 2.2 Collagen‐induced arthritis model

The well‐established rat collagen‐induced arthritis (CIA) method was used to induce arthritis in the RA group, as previously described (Asquith et al., 2009; Kannan et al., 2005; Oyenihi et al., 2019; Song et al., 2015). Briefly, bovine heterologous type II collagen (Chondrex Inc., WA, USA) was dissolved in 0.01N glacial acetic acid (2 mg/ml), followed by the preparation of an emulsion using an equal volume of incomplete Freund's adjuvant (Chondrex Inc., WA, USA). The emulsion was injected intra‐dermally twice just above the tail region of each rat under isoflurane anesthesia, 7 days apart. Non‐arthritis control rats were subjected to identical anesthesia, but were not subjected to any injections. The peak of the acute disease in terms of immune response typically occurs at 3 weeks (acute disease) and maximal joint damage occurs at 5 weeks (Rajaiah and Moudgil., 2009), therefore 5 weeks post‐induction was selected as sample collection time point for the current study. Therefore, after a 5‐week experimental period, all rats were killed by guillotine decapitation. The *gastrocnemius* muscle was removed, weighed, and frozen in liquid nitrogen‐cooled isopentane and then stored at −80°C until subsequent analysis. This muscle was previously shown to be most severely affected by CIA, with fiber type not a major determinant of cachexia outcome (Oyenihi et al., 2019).

Successful induction of arthritis was confirmed by anti‐collagen antibody titer testing, as well as clinical symptoms (e.g., paw edema) as previously described (Oyenihi et al., 2019). All 10 animals treated with incomplete Freund's adjuvant developed arthritis symptoms. Neither the date of onset of observable symptoms such as edema, nor symptom severity (e.g., the number of limbs affected), correlated with antibody titer, and was highly variable between individuals. Therefore, we did not employ a selection protocol based on observed symptoms, in an attempt to account for the inter‐individual variation seen in RA. The full details of the confirmation tests employed have been published earlier (Oyenihi et al., 2019). The muscle samples used for generation of data presented here, were obtained from the cited study, which was previously conducted in our group, but all data presented here are novel (i.e., there is no duplication of previously published data).

### 2.3 Muscle histology

Frozen *gastrocnemius* tissues were sectioned into 10 μm cross‐sections using a cryostat (Leica CM1860 UV, Leica Biosystems Nussloch GmbH, Germany) at −25°C. To ensure consistency between samples, a predetermined, standardized section was cut off the proximal end of each sample before sectioning, so that all sections were obtained at a similar locality (depth and distance from proximal end) within the muscle. This allowed sections from all samples to come from the center of the muscle.

#### 2.3.1 Cell populations and cross‐sectional area

Tissue sections were fixed in 4% paraformaldehyde (158127, Sigma‐Aldrich, USA), washed and blocked for 90 minutes in 5% donkey serum (S217G, Celtic Diagnostics, South Africa) in 1% bovine serum albumin (BSA, 10735086001, Roche, Germany), with 0.2% Triton‐X‐100 (X100, Sigma‐Aldrich, USA). Sections were incubated with primary antibodies for Pax7 (1:50, Pax7, Dev. Studies Hybridoma Bank, USA), F4/80 (1:200, sc377009, Lot#I1317, Santa Cruz, USA), and α‐smooth muscle actin (1:250, α‐SMA, Lot#125M4797V, A2547, Sigma‐Aldrich, Germany) in 1% BSA overnight at 4°C. After washing, secondary antibodies were added for 2 hours (Donkey Anti‐mouse 555, 1:500, ab150110, Abcam). Tissues were stained with Agglutinin (W11261, Thermo Fisher Scientific, USA) and Hoechst stain (ab33342, Abcam, UK).

Imaging was performed on a Confocal Microscope (Carl Zeiss LSM 780, Zeiss, Germany) and analyzed on Zen 2011 software (Zeiss, Germany). The EC Plan‐Neofluar 10x/0.3 M27 and Plan‐Apochromat 20x/0.8 M27 objectives were used to acquire images at 100x and 200x magnification, respectively. (Magnification represented as ocular lens (10x) multiplied by objective lens (10x/20x)).

#### 2.3.2 Image analysis

For each animal, two cryosections were prepared, with one field of view analyzed per section. (Both image acquisition and image analysis were performed on blinded samples.) Using ImageJ software (version 1.49, Wayne Rasband), the CSA of 50 fibers per sample was measured using the free‐hand outline tool. The cell counter tool was used to count the number of positively stained macrophages, satellite cells, myofibroblasts, myonuclei, as well as the number of fibers per field of view. Cells were determined by a positive stain of both the antibody and Hoechst. Images acquired at 100x magnification were used for CSA measurements and images acquired at 200x magnification were used for all cell counts.

### 2.4 Cytokine and growth factor analysis

Thirty to forty milligrams of muscle tissue was added to RIPA‐based lysis buffer (containing 2X Complete Protease Inhibitor, 1X Complete Phosphatase Inhibitor, Roche) and homogenized using the PolyTron Manual Disperser (Kinematica, AG). After centrifugation, the supernatant was removed and used as the final protein sample. Protein concentrations were determined with a commercial Bicinchoninic acid (BCA) kit (BCA protein assay, Thermo Fischer Scientific, USA) according to the manufacturers’ guidelines. Twenty microgram of protein from each sample was used for electrophoresis on polyacrylamide gels consisting of a 12% separating gel (0.39 M Tris‐HCl (pH 8.8), 30% Acrylamide, 1% SDS, 1% APS, 0.07% TEMED) and a 4% stacking gel (0.125 M Tris‐HCl (pH 6.8), 12.5% Acrylamide, 1% SDS, 1% APS, 0.1% TEMED). Post electrophoresis gels were transferred onto a nitrocellulose membrane (GE Healthcare, Life science, RPN 3032D, UK) via a Turbo‐blot transfer system (Bio‐Rad, USA). After blocking for 1 hr with either 5% fat‐free milk in TBS‐T or 1% BSA‐TBS‐T, membranes were incubated with primary antibodies in either 5% fat‐free milk‐TBS‐T or 1% BSA (Bovine Serum Albumin Fraction V, Roche, USA) in 1xTBS‐T at 4°C overnight. Primary antibodies included TNF‐α (1:1000, NB600‐587, Lot#41630, Novus Biologicals), IL‐1β (1:1000, NB600‐633, Lot#33815, Novus Biologicals), IL‐6 (1:1000, NB600‐1131, Lot#38801, Novus Biologicals), NFκB (1:1500, ab16502, Lot#GR3266473‐1, Abcam), IL‐10 (1:1000, ab9969, Lot#GR40933‐42, Abcam), Id2 (1:1000, ab166708, Lot#GR3364842‐1, Abcam), Myogenin (1:1000, sc12732, Lot#G0510, Santa Cruz), MyoD (1:1500, m3512, Lot#10045966, DAKO), PCNA (1:1000, ab15497, Lot#GR305622‐1, Abcam), Mafbx (1:1500, ab168372, Lot#GR3241581‐6, Abcam), TGF‐β (1:800, ab92486, Lot#GR312172‐2, Abcam), bone morphogenetic protein‐7 (BMP‐7, 1:1000, NBP1‐69126, Lot#QC1792‐42005, Novus Biologicals), MIF (1:1000, ab65869, Lot#GR221952‐35, Abcam), MCP‐1 (1:1500, NBP1‐07035, Lot#B‐3, Novus Biologicals), and GAPDH (1:4000, ab9485, Lot#GR3243682‐1, Abcam). Membranes were incubated with horseradish peroxidase (HRP)‐linked secondary antibody (Cell Signaling, 7074S, Lot#26 or 7076S, Lot#31) 1:15,000 in 5% fat‐free milk in TBS‐T for 1 hour and reaction with enhanced chemiluminescence (ECL, SuperSignal West Femto Maximum Sensitivity Substrate, 34094, Thermo Scientific) enabled subsequent imaging using the Chemidoc MP and Image Lab software. Immunoreactive proteins were then quantified using the same software and were normalized against GAPDH staining and the reference sample. Selection of antibodies was performed after confirmation of specificity, based on specifications provided by manufacturers. Secondary antibodies were also validated by the absence of nonspecific binding. All proteins were detected at the predicted molecular weight.

### 2.5 Statistical analysis

Statistical analysis was performed on GraphPad Prism v.8. Shapiro–Wilk test for normality was used to determine whether parametric or nonparametric tests were required. T‐tests were performed for comparison between groups (unpaired t‐test for normally distributed data; Mann–Whitney test for nonparametric data). Correlation analysis was performed (Pearson correlation coefficient or Spearman rank for parametric and nonparametric data, respectively). *p *< 0.05 was considered statistically significant.

## 3 RESULTS

**FIGURE 1:**
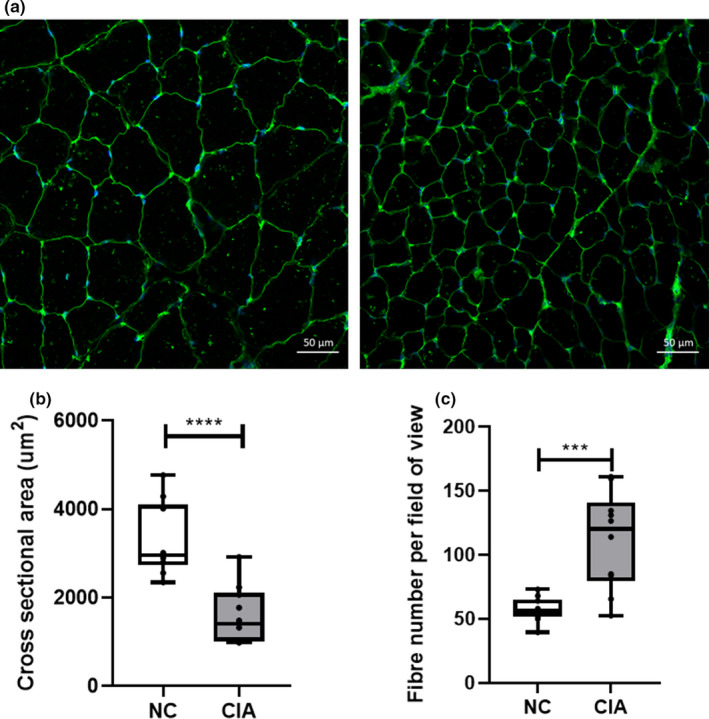
Representative immunofluorescent images (a) and quantitated data (b) comparing cross‐sectional areas of non‐arthritic control (NC) versus collagen‐induced arthritis (CIA) *gastrocnemius* muscle. *n* = 10 per group. 100x magnification. Scale bar represents 50 µm. Statistical analysis: Unpaired *t*‐test. *p *< 0.0001. Data represented as box and whisker plots indicating the highest and lowest values, the median and the interquartile range, as well as individual data points

*Gastrocnemius* muscle of rodents subjected to CIA exhibited a 57% reduction in CSA (*p* < 0.0001) when compared with non‐arthritic controls (Figure 1).

**FIGURE 2:**
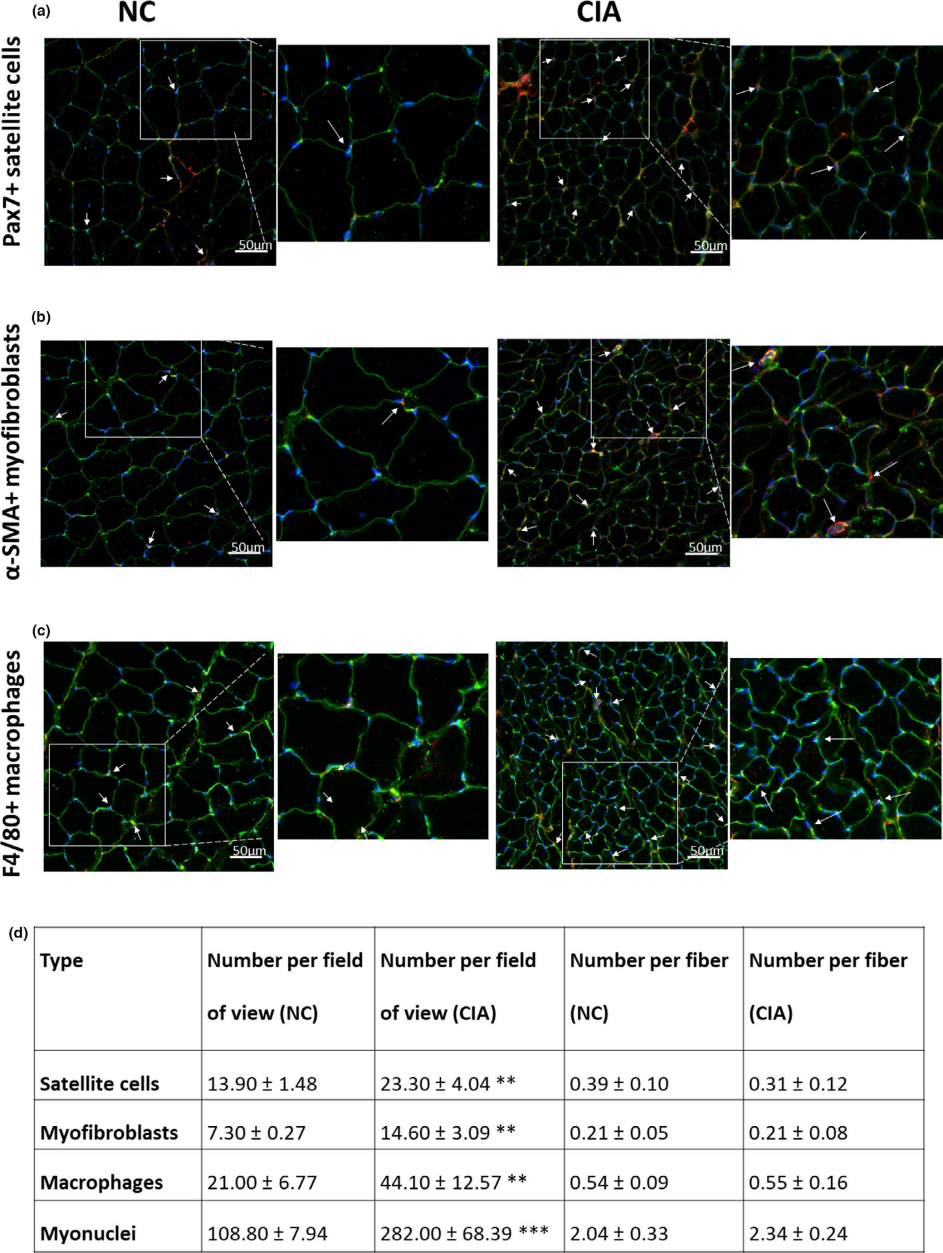
Representative images (a–c) and quantification of (d) satellite cells, myofibroblasts (including myonuclei number) and macrophages in control and CIA rodent *gastrocnemius* muscle, expressed both as average number of cells per field of view and average number of cells per myofiber. *n* = 5 per group. Statistical analysis: Unpaired *t*‐test (normally distributed) or Mann–Whitney test (not normally distributed). **, *p *< 0.01; ***, *p *< 0.001 when compared with non‐arthritic controls. Data expressed as mean ±SD. Magnification 200x. NC, non‐arthritic control; CIA, collagen‐induced arthritis. Western blot analysis of intramuscular cytokine levels (Figure 3) indicated an increase in proinflammatory and a decrease in anti‐inflammatory cytokines in the CIA rodent muscle compared to non‐arthritic controls. Specifically, there was a significant increase in TNF‐α (*p *< 0.01), whereas there was a significant reduction in IL‐10 (*p *< 0.05). The other proinflammatory cytokines did not exhibit significant changes, this included IL‐1β (*p *= 0.21), IL‐6 (*p *= 0.17), and NFκB (*p *= 0.14). A noteworthy observation was that CIA did not seem to have an effect on levels of MCP‐1 (*p *= 0.71) or MIF (*p *= 0.72), cytokines specifically associated with macrophage migration into tissue

**FIGURE 3:**
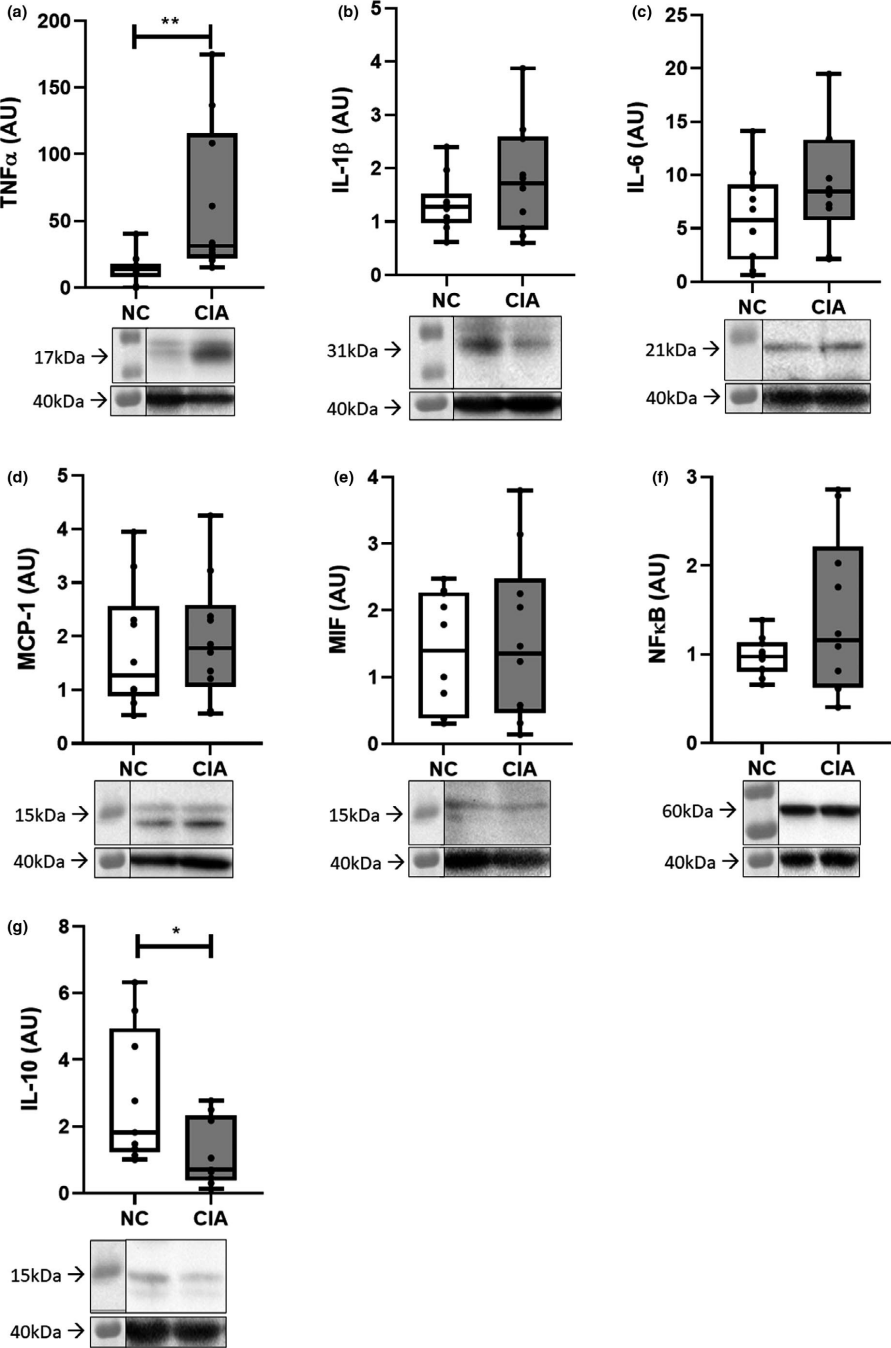
Intramuscular pro and anti‐inflammatory cytokine levels in *gastrocnemius* muscle from normal control versus collagen‐induced arthritis rodents. (a) TNFα; (b) IL‐1β; (c) IL‐6; (d) MCP‐1; (e) MIF; (f) NFκB; (g) IL‐10. *n *= 10 per group. Statistical analysis: Unpaired t‐test (parametric for b–f) or Mann–Whitney test (nonparametric for a, g). *, *p *< 0.05; **, *p *< 0.01. Data represented as box and whisker plots indicating the highest and lowest values, the median and the interquartile range, as well as individual data points. TNF‐α, tumor necrosis factor‐α; IL‐1β, interleukin‐1β; MCP‐1, monocyte chemoattractant protein; MIF, macrophage migration inhibitory factor; NF‐κB, Nuclear Factor kappa‐light‐chain‐enhancer of activated B cells. Representative images are provided for the protein of interest (top) and housekeeping protein (GAPDH, bottom) under each graph

Absolute counts of intramuscular abundance of cells and nuclei (Figure 2) revealed significant increases in the number of satellite cells, myofibroblasts, macrophages (*p *< 0.01), and myonuclei (*p *< 0.001) per field of view in the CIA *gastrocnemius* when compared with non‐arthritic controls. When expressing cell and myonuclei counts relative to number of myofibers per field of view, the CIA‐associated increases were no longer evident (Figure 3).

**FIGURE 4:**
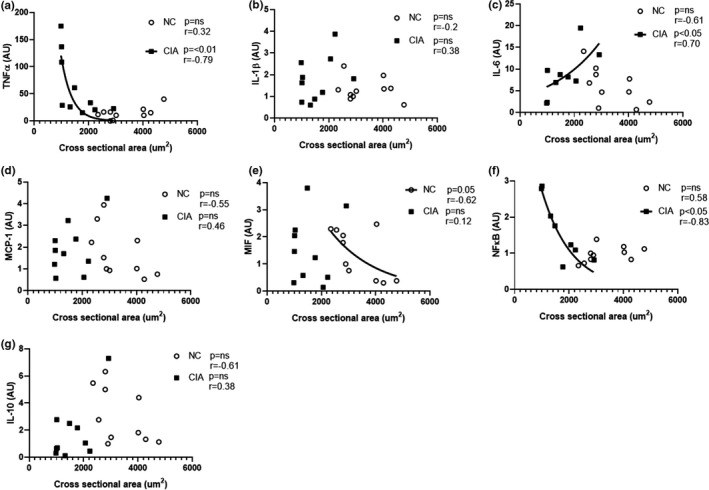
Correlation between intramuscular cytokine levels and cross‐sectional area of the *gastrocnemius* muscle in non‐arthritic control (NC) and collagen‐induced arthritic (CIA) rats – (a) TNFα; (b) IL‐1β; (c) IL‐6; (d) MCP‐1; (e) MIF; (f) NFκB; (g) IL‐10. *n* = 10 per group. Statistical analysis: Pearson linear correlation (parametric for b–f) or Spearman correlation (nonparametric for a, g). TNF‐α, tumor necrosis factor‐α; IL‐1β, interleukin‐1β; MCP‐1, monocyte chemoattractant protein; MIF, macrophage migration inhibitory factor; NF‐κB, Nuclear Factor kappa‐light‐chain‐enhancer of activated B cells

The observation of relatively large interindividual variability in cytokine levels as well as in the CSA of the muscle fibers prompted further investigation of the correlation between cytokine levels and extent of muscle cachexia. Both TNF‐α and NFκB (*p *< 0.01 and *p *< 0.05) demonstrated a significant negative correlation to CSA in CIA rodents, whereby a smaller fiber CSA was correlated to a higher cytokine level. The opposite was demonstrated for IL‐6 (*p *< 0.05) where greater fiber CSA was correlated with higher cytokine levels (Figure 4c). Additionally, MIF was negatively correlated with CSA (*p *= 0.05) in NC rodents, but not in the CIA rodents (Figure 4e). There was no significant correlation to CSA in the remaining NC samples.

**FIGURE 5:**
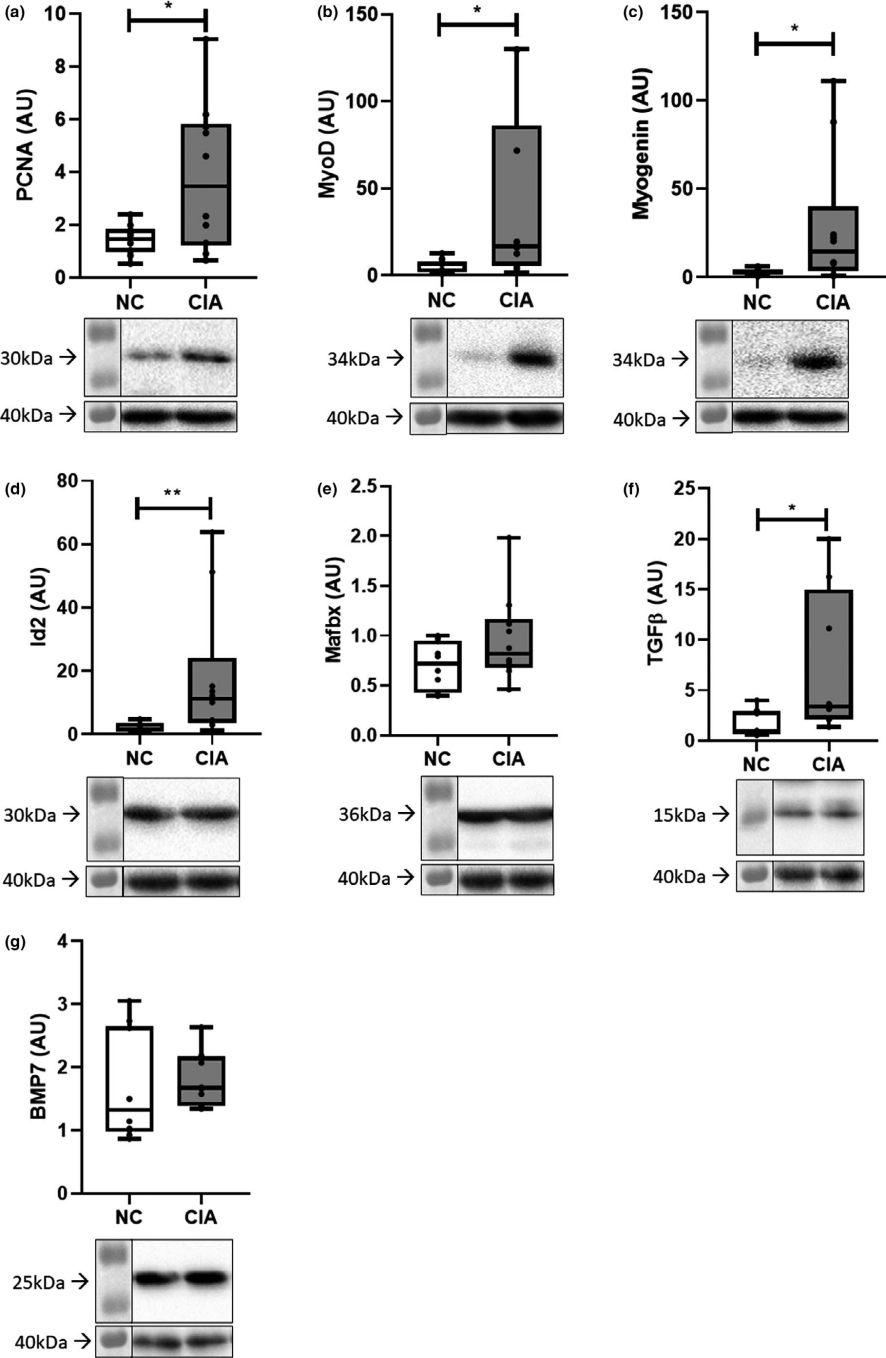
Muscle proliferation and differentiation markers in control vs CIA gastrocnemius muscle. (a) PCNA; (b) MyoD; (c) Myogenin; (d) Id2; (e) Mafbx; (f) TGFβ; (g) BMP‐7. *n* = 10 per group. Statistical analysis: Unpaired *t*‐test (parametric for a, e) or Mann–Whitney test (nonparametric for b–d, f, g). *, *p *< 0.05; **, *p *< 0.01. Data represented as box and whisker plots indicating the range, the median and the 25–75 interquartile range, as well as individual data points. PCNA, proliferating cell nuclear antigen; TGFβ, transforming growth factor‐β; Id2, inhibitor of differentiation; Mafbx, muscle atrophy f‐box; BMP‐7, bone morphogenetic protein. Representative images are provided for the protein of interest (top) and housekeeping protein (GAPDH, bottom) under each graph

CIA was associated with a significantly higher expression of the markers indicative of cell proliferation, PCNA and MyoD when compared with controls (both *p *< 0.05; Figure 5a,b). In addition, differentiation marker myogenin was significantly higher (*p *< 0.05; Figure 5c) in CIA tissue as well as the growth factor TGFβ (*p *< 0.05; Figure 5f). In contrast, CIA muscle exhibited higher levels of Id2, an inhibitor of differentiation, when compared with controls (*p *< 0.01; Figure 5d). Mafbx, a marker of muscle wasting, seemed unaffected in CIA (Figure 5e). BMP‐7, a stimulator of muscle growth, remained unchanged (Figure 5g).

**FIGURE 6:**
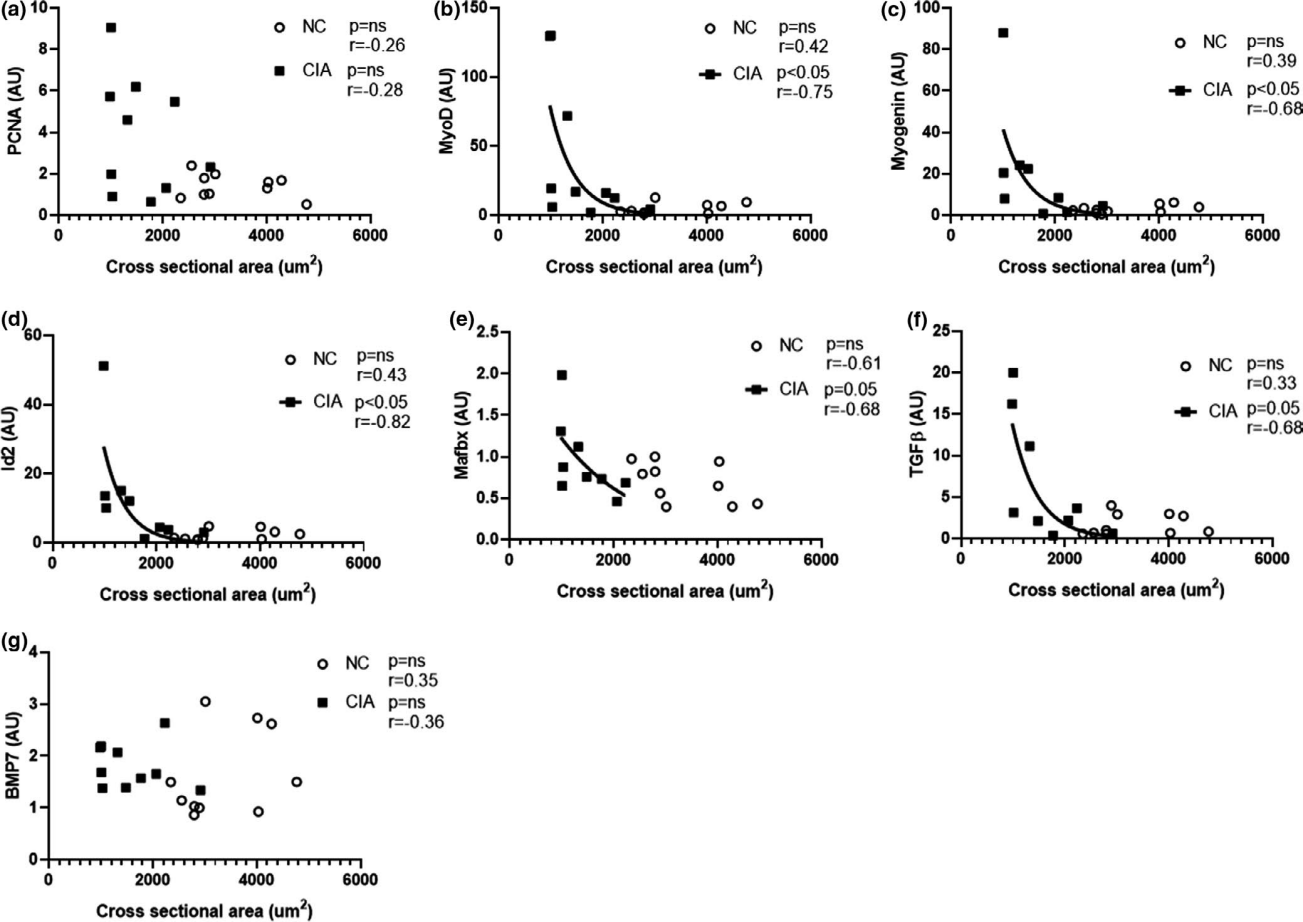
Correlation between proliferation and differentiation markers and cross‐sectional area of the *gastrocnemius* muscle. (a) PCNA; (b) MyoD; (c) Myogenin; (d) Id2; (e) Mafbx; (f) TGFβ; (g) BMP‐7. *n* = 9–10 per group. Statistical analysis: Pearson correlation (parametric for a, e, g) or Spearman correlation (nonparametric for b–d, f). PCNA, proliferating cell nuclear antigen; TGFβ, transforming growth factor‐β; Id2, inhibitor of differentiation; Mafbx, muscle atrophy f‐box; BMP‐7, bone morphogenetic protein

Similar to correlations observed between cytokine levels and muscle fiber CSA, CSA also correlated with most muscle growth factors in the CIA rodents; with the exception of PCNA (which exhibited high variability) and BMP‐7, all parameters assessed correlated negatively with CSA (Figure 6). This differed to the NC rodents, of which none were significantly correlated to CSA.

**FIGURE 7:**
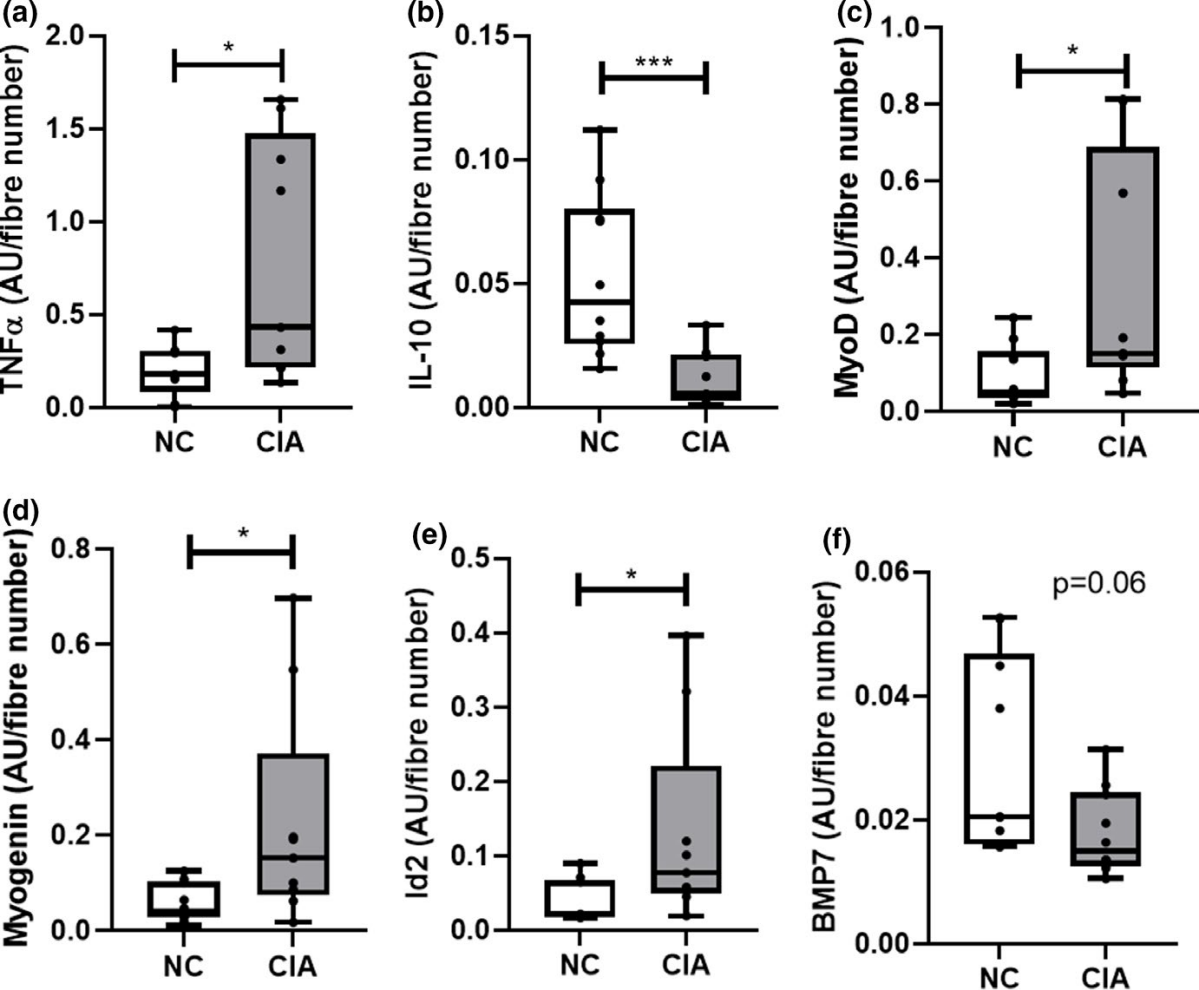
Comparison of cytokines and growth factors taking fiber number into consideration. (a) TNF‐α; (b) IL‐10; (c) BMP7; (d) Id2; (e) MyoD; (f) Myogenin. *n* = 10 per group. Statistical analysis: Mann–Whitney test. *, *p *< 0.05; **, *p *< 0.01. Data represented as box and whisker plots indicating the highest and lowest values, the median and the interquartile range, as well as individual data points. TNF‐α, tumor necrosis factor‐α; IL‐10, interleukin‐10; BMP‐7, bone morphogenetic protein; Id2, inhibitor of differentiation

Taking into consideration the data from Figure 2 which indicates an increased fiber number per area of muscle, and the fact that RA‐associated decreases in muscle fiber CSA did not appear to affect satellite cell number per fiber, it may be relevant to express parameters reported here, relative to the number of nuclei available to receive these signals. As an analysis by western blot does not account for fiber or cell number, we also expressed data for cytokines and growth factors relative to fiber number. When data were expressed relative to fiber number, many CIA‐associated differences were no longer observed (Figure S1). However, in the context of inflammation, TNF‐α remained significantly elevated and IL‐10 significantly decreased in CIA rodents (Figure 7a,b). Furthermore, BMP‐7 demonstrated a new tendency for a decreased expression in CIA (Figure 7c), whereas muscle growth and differentiation markers, MyoD and myogenin, as well as the differentiation inhibitor Id2, remained significantly elevated (Figure 7d–f).

## 4 DISCUSSION

The current study assessed intramuscular cytokines in a rodent CIA model as a representative model of human rheumatoid cachexia. Research into the intramuscular cytokine concentrations in rheumatoid arthritis and CIA is extremely limited, with only one other paper, to our knowledge, measuring these levels in RA patients (Huffman et al., 2017). Associated parameters assessed include downstream signaling and myogenic regulation. The novel research data presented here, highlights firstly the importance of assessing both TNF‐α and IL‐10 as indicators of the severity of this RA‐induced co‐morbidity. Secondly, current data suggest that expression of data relative to fiber number per area (as an indirect indicator of signaling to nuclei of all other cell types involved, and as an indicator of signaling affecting the fibers themselves) may provide a more realistic picture of the multi‐cellular signaling occurring in RA‐affected skeletal muscle.

In terms of the severity of cachexia demonstrated in the CIA model, immunofluorescence analysis of muscle cross sections demonstrated a significant reduction in CSA. This is in line with data reported by other groups in both the rodent CIA (Filippin et al., 2013; Horai et al., 2013) and human RA models (Helliwell & Jackson, 1994; Matschke et al., 2010), as well as with a previous report from our group, demonstrating an overall left shift in fiber size distribution in multiple muscle types (Oyenihi et al., 2019). This, along with the loss in muscle mass itself, is one of the defining characteristics of cachexia, an occurrence in both early‐ and late‐stage RA.

Assessment of cellular profile changes in RA muscles is also lacking, with early studies merely indicating the presence of inflammatory infiltrate and fibrosis but not measuring the extent of cell type involvement (Finol et al., 1988). Here, we demonstrate an overall increased presence of satellite cells, macrophages, myofibroblasts, and myonuclei per volume of CIA *gastrocnemius* muscle when compared with controls. However, this difference is not maintained when considering the numbers of these cells on a “per fiber” basis. The satellite cell data corresponds with a study of human RA‐affected muscle biopsies, where satellite cell number per fiberwas also not increased in response to the disease (Boutrup et al., 2018). Current data suggest that counting of cells in the muscle tissue does not give the full picture and the signaling molecules should be taken in account to measure the processes predominating in the muscle.

It is widely known that pro‐inflammatory cytokines, such as TNF‐α and IL‐1β, play a role in the pathophysiology of RA to the extent that a number of therapies have been designed to solely target these cytokines (Brennan & McInnes, 2008; Dayer, 2003). However, an investigation into these cytokines within the muscle of rheumatoid cachexia models is limited, with most studies detecting increases in plasma or synovial fluid pro‐inflammatory cytokines and reductions in anti‐inflammatory cytokines (Roubenoff et al., 1994). It has also been suggested that TNF‐α plays a role in triggering inflammation locally and systemically, whereas IL‐1β is more involved in local processes of cartilage and bone destruction (Dayer, 2002), another reason why IL‐1β may remain low in muscle tissue compared to TNF‐α. In response to an insult, TNF‐α is released by circulating macrophages, which further stimulate the production and release of more TNF‐α as well as IL‐1. IL‐1 then induces the production of IL‐6 (Ott et al., 2007). The significant increase in TNF‐α in the current study indicates the presence of active disease and the central role of TNF‐α. Further highlighting the importance of TNF‐α in the development of rheumatoid cachexia, it remains elevated when correcting data for fiber number per area of muscle, confirming the availability of relatively more TNF‐α signaling per fiber and thus per macrophage, satellite cell, and fibroblast. In addition, IL‐10 emerges as another robust indicator of inflammatory status in arthritis muscle, while arthritis‐ascribed differences in other pro‐inflammatory cytokines disappeared when expressing data on a per fiber basis. In human RA, assessment of intramuscular cytokines has demonstrated elevated IL‐6 concentrations in RA patients, with other cytokines not showing significant changes (Huffman et al., 2017). Of particular interest, the study by Huffman et al., (2017) determined that muscle cytokine levels did not correspond to cytokine levels in circulation, highlighting the importance of measuring intramuscular cytokine concentrations when assessing rheumatoid cachexia. In the current study, CSA and IL‐6 showed a significant positive correlation in CIA. IL‐6, as a myokine has exhibited both pro‐ and anti‐inflammatory effects within the muscle (Muñoz‐Cánoves et al., 2013), as well as exhibiting both hypertrophic effects through the stimulation of satellite cell proliferation (Serrano et al., 2008), and the promotion of atrophy. However, atrophy as a result of IL‐6 is largely dose‐ and time‐dependent (Haddad et al., 2005). IL‐6 would seem to correlate with a hypertrophy outcome in the current study. Indeed, it has been suggested that IL‐6 is the predominant cytokine present in the later maintenance stage of RA compared to TNF‐α in the earlier stages (Kung et al., 2020) However, a longitudinal assessment is probably required before firm interpretation can be made.

In terms of muscle growth factors, there was an overall increase in proliferation markers PCNA and the early myogenic marker, MyoD, as well as the differentiation marker, myogenin, in the CIA *gastrocnemius* muscle indicating an attempt to repair damaged muscle or promote regrowth following atrophy. This has also been demonstrated at different stages of atrophy in another rodent study (Castillero et al., 2009). MyoD and myogenin remain elevated when taking into consideration the fiber number, highlighting that these two proteins have an exaggerated effect in attempting to repair the muscle despite satellite cell numbers remaining the same. However, in the CIA tissue, Id2, a marker for inhibition of differentiation is also significantly upregulated. Due to its ability to bind to and inhibit MyoD and other myogenic regulatory factors, Id2 may be contributing significantly to the poor muscle outcomes seen in cachexia (Jen et al., 1992; Liu et al., 2002). However, alongside its actions as a negative regulator of the myogenic regulatory factor family, Id2 has also been implicated in apoptosis‐related atrophy (Alway et al., 2003), a factor that requires more elucidation in rheumatoid cachexia. Once again, these markers, along with the atrogen, Mafbx, showed a negative correlation to CSA. Overall, we suggest that the damage to the muscle due to inflammatory infiltration and fibrosis (Oyenihi et al., 2019), and increased muscle breakdown, all contribute to necrosis and muscle loss, which is then followed by an attempt for muscle repair to occur with increased proliferation and differentiation of satellite cells. However, differentiation is also being strongly inhibited resulting in poor regenerative ability, leading to smaller fibers. As severity of cachexia is increased, more repair is attempted and failed, as indicated by the correlation to CSA. In addition to this, the increased inflammation itself may lead to an increase in the proliferation of myoblasts within the muscle (Bencze et al., 2012), whereas the reduced anti‐inflammatory cytokines contribute to the reduced differentiation and regeneration (Arnold et al., 2007), ultimately leading to smaller fibers. Additionally, TNF‐α, which is significantly increased in this study, has been shown to stimulate the ubiquitin‐proteasome pathway (Li et al., 2005; López‐Menduiña et al., 2010). Both TNF‐α and Mafbx follow the same trend in terms of correlation to CSA which may suggest that they are some of the many components working together in the development of cachexia, it possible that in this study, TNF‐α is elevated first, and the increase in Mafbx may reach significance at a later stage in disease development. It is also possible that Mafbx is upregulated at earlier stages in the development of cachexia as a study on rat CIA indicated peaked Mafbx at day 15 (Castillero et al., 2009). More information may be obtained from a longitudinal study design, which may shed more light on dynamic changes over the course of disease progression. BMP‐7 is vital for the maintenance of muscle mass after disruption of the neuromuscular junction, and contributes to hypertrophy (Winbanks et al., 2013). In the *gastrocnemius* of the CIA rodent, BMP‐7 does not change, nor is it correlated to CSA. However, when taking fiber number into consideration, there is a trend for a decrease in BMP‐7, highlighting another factor that may be acting directly on the muscle fibers to influence rheumatoid cachexia.

In addition to its ability to inhibit muscle differentiation and contribute to apoptosis‐related atrophy, Id2 also plays a role in the regulation of fibrosis. Id2 enhances the proliferation of various cell types but also mediates TGFβ (Izumi et al., 2006; Yin et al., 2019). While TGFβ is vital in muscle growth via satellite cell activation (Delaney et al., 2017), it also largely contributes to the production of collagen to develop fibrosis. Here, TGFβ is significantly increased compared to normal controls, and follows the same negative correlation as many of the other proteins when compared with CSA. We suggest that Id2 may be contributing to the proliferation of a variety of cells, including myoblasts and fibroblasts, and may be regulated in combination with TGFβ. However, TGFβ appears to predominate based on the continued increase and on the fibrotic content seen in a previous study (Oyenihi et al., 2019). Additionally, BMP‐7, a TGF‐β antagonist, exerts anti‐fibrotic effects (Wu & Hatzopoulos, 2019), a mechanism which may be lost by its reduction in skeletal muscle. BMP‐7 also reduced monocyte infiltration and increased M2 macrophage concentrations and anti‐inflammatory cytokine levels in a rodent model of carotid artery ligation (Singla et al., 2016). Considering its muscle growth and anti‐fibrotic effects, as well as these potentially positive effects on inflammation, it may be useful to investigate BMP‐7 as a potential treatment option in the context of rheumatoid cachexia.

## 5 CONCLUSION

Novel data reported here include the first comprehensive assessment of muscle regenerative capacity and intramuscular cytokine profile in CIA. In addition, current data illustrate for the first time, the cellular profile of CIA muscle.

Based on the increases in proliferation and differentiation markers, data suggest that CIA‐associated cachexic muscle remains capable of regeneration, and that the unresolved inflammation is the primary role player impairing the maintenance of muscle fiber CSA. In terms of identification of therapeutic targets, current data do not support an approach focusing on modulation of muscle regenerative capacity *per se*, and rather indicate that the primary focus should remain on the resolution of the chronic inflammation itself. In this respect, data further suggest that IL‐10 should be assessed in addition to TNF‐α, as indicators of inflammation severity. On a more practical note, data presented suggest that expressing data on a per fiber basis, may present a clearer picture of the signaling events at a cellular level.

## Conflicts of interest

The authors declare that they have no conflict of interest.

## AUTHOR CONTRIBUTIONS

Conceptualization was jointly performed by all TO, KHM, and CS. YSLP executed the CIA protocol and performed sample collection. The literature search, sample analysis, data reduction, and first draft were performed/written by TO, under the supervision of CS. All authors revised the manuscript. All authors read and approved the final manuscript.

## Supporting information



Supplementary Material
