# Supplementary material for: Unresolved intramuscular inflammation, not diminished skeletal muscle regenerative capacity, is at the root of rheumatoid cachexia: insights from a rat CIA model
Source: Physiol Rep. 2021 Nov 21;9(22):e15119. doi: 10.14814/phy2.15119 (PMC8606867; doi:10.14814/phy2.15119)
Supplement: Supplementary file 1 — Supplementary Material [file PHY2-9-e15119-s001.docx]

Supplementary Data

Supplementary Table 1: Correlation comparisons for protein expression compared to cross-sectional area, alone and taking fiber number into account.

| variable 1 | variable 2 | Pearson | Pearson p-val | Spearman | Spearman p-val | # cases | NC/CIA |
| --- | --- | --- | --- | --- | --- | --- | --- |
| TNF-a (AU) | CSA | -0,5 | 0,03 | -0,38 | 0,09 | 20 |  |
| IL-1ß (AU) | CSA | -0,14 | 0,55 | -0,08 | 0,75 | 20 |  |
| BMP7 (AU) | CSA | 0,01 | 0,95 | -0,14 | 0,55 | 20 |  |
| IL-10 (AU) | CSA | 0,24 | 0,3 | 0,38 | 0,1 | 20 |  |
| IL-6 (AU) | CSA | -0,27 | 0,25 | -0,22 | 0,36 | 20 |  |
| MCP-1 (AU) | CSA | -0,14 | 0,56 | -0,16 | 0,51 | 20 |  |
| MIF (AU) | CSA | -0,21 | 0,38 | -0,08 | 0,74 | 20 |  |
| Id2 (AU) | CSA | -0,56 | 0,01 | -0,6 | <0.01 | 20 |  |
| Mafbx (AU) | CSA | -0,52 | 0,02 | -0,52 | 0,02 | 20 |  |
| NFκB (AU) | CSA | -0,36 | 0,12 | -0,11 | 0,64 | 20 |  |
| pSmad1/5/8 (AU) | CSA | -0,14 | 0,56 | -0,13 | 0,58 | 20 |  |
| MyoD (AU) | CSA | -0,53 | 0,02 | -0,52 | 0,02 | 20 |  |
| Myogenin (AU) | CSA | -0,53 | 0,02 | -0,54 | 0,02 | 20 |  |
| PCNA (AU) | CSA | -0,53 | 0,02 | -0,42 | 0,07 | 20 |  |
| TGFß (AU) | CSA | -0,5 | 0,03 | -0,38 | 0,09 | 20 |  |
| TNF-a (AU/fiber no.) | CSA | -0,38 | 0,09 | -0,3 | 0,2 | 20 |  |
| IL-1ß (AU/fiber no.) | CSA | 0,41 | 0,07 | 0,52 | 0,02 | 20 |  |
| BMP7 (AU/fiber no.) | CSA | 0,71 | <0.01 | 0,7 | <0.01 | 20 |  |
| IL-10 (AU/fiber no.) | CSA | 0,38 | 0,1 | 0,62 | <0.01 | 20 |  |
| IL-6 (AU/fiber no.) | CSA | 0,11 | 0,66 | 0,15 | 0,52 | 20 |  |
| MCP-1 (AU/fiber no.) | CSA | 0,23 | 0,34 | 0,37 | 0,11 | 20 |  |
| MIF (AU/fiber no.) | CSA | 0,16 | 0,5 | 0,26 | 0,27 | 20 |  |
| Id2 (AU/fiber no.) | CSA | -0,49 | 0,03 | -0,47 | 0,04 | 20 |  |
| Mafbx (AU/fiber no.) | CSA | 0,37 | 0,11 | 0,35 | 0,13 | 20 |  |
| NFκB (AU/fiber no.) | CSA | 0,64 | <0.01 | 0,51 | 0,02 | 20 |  |
| pSmad1/5/8 (AU/fiber no.) | CSA | 0,24 | 0,3 | 0,29 | 0,22 | 20 |  |
| MyoD (AU/fiber no.) | CSA | -0,44 | 0,06 | -0,34 | 0,14 | 20 |  |
| Myogenin (AU/fiber no.) | CSA | -0,47 | 0,04 | -0,38 | 0,1 | 20 |  |
| PCNA (AU/fiber no.) | CSA | -0,14 | 0,54 | -0,09 | 0,7 | 20 |  |
| TGFß (AU/fiber no.) | CSA | -0,27 | 0,25 | -0,16 | 0,5 | 20 |  |
| TNF-a (AU) | CSA | 0,14 | 0,7 | 0,33 | 0,35 | 10 | NC |
| IL-1ß (AU) | CSA | -0,2 | 0,57 | -0,1 | 0,79 | 10 | NC |
| BMP7 (AU) | CSA | 0,33 | 0,35 | 0,35 | 0,33 | 10 | NC |
| IL-10 (AU) | CSA | -0,51 | 0,13 | -0,61 | 0,07 | 10 | NC |
| IL-6 (AU) | CSA | -0,61 | 0,06 | -0,7 | 0,03 | 10 | NC |
| MCP-1 (AU) | CSA | -0,55 | 0,1 | -0,65 | 0,05 | 10 | NC |
| MIF (AU) | CSA | -0,62 | 0,06 | -0,6 | 0,07 | 10 | NC |
| Id2 (AU) | CSA | 0,42 | 0,23 | 0,43 | 0,22 | 10 | NC |
| Mafbx (AU) | CSA | -0,52 | 0,13 | -0,61 | 0,07 | 10 | NC |
| NFκB (AU) | CSA | 0,42 | 0,23 | 0,58 | 0,09 | 10 | NC |
| pSmad1/5/8 (AU) | CSA | 0,06 | 0,86 | 0,25 | 0,49 | 10 | NC |
| MyoD (AU) | CSA | 0,44 | 0,2 | 0,42 | 0,23 | 10 | NC |
| Myogenin (AU) | CSA | 0,57 | 0,09 | 0,39 | 0,26 | 10 | NC |
| PCNA (AU) | CSA | -0,26 | 0,47 | -0,12 | 0,76 | 10 | NC |
| TGFß (AU) | CSA | 0,14 | 0,7 | 0,33 | 0,35 | 10 | NC |
| TNF-a (AU/fiber no.) | CSA | 0,69 | 0,03 | 0,55 | 0,1 | 10 | NC |
| IL-1ß (AU/fiber no.) | CSA | 0,05 | 0,89 | -0,05 | 0,89 | 10 | NC |
| BMP7 (AU/fiber no.) | CSA | 0,6 | 0,07 | 0,41 | 0,25 | 10 | NC |
| IL-10 (AU/fiber no.) | CSA | -0,4 | 0,25 | -0,41 | 0,25 | 10 | NC |
| IL-6 (AU/fiber no.) | CSA | -0,53 | 0,12 | -0,7 | 0,03 | 10 | NC |
| MCP-1 (AU/fiber no.) | CSA | -0,44 | 0,21 | -0,54 | 0,11 | 10 | NC |
| MIF (AU/fiber no.) | CSA | -0,54 | 0,11 | -0,53 | 0,12 | 10 | NC |
| Id2 (AU/fiber no.) | CSA | 0,63 | 0,05 | 0,48 | 0,17 | 10 | NC |
| Mafbx (AU/fiber no.) | CSA | -0,22 | 0,54 | -0,42 | 0,23 | 10 | NC |
| NFκB (AU/fiber no.) | CSA | 0,77 | <0.01 | 0,72 | 0,02 | 10 | NC |
| pSmad1/5/8 (AU/fiber no.) | CSA | 0,24 | 0,5 | 0,53 | 0,12 | 10 | NC |
| MyoD (AU/fiber no.) | CSA | 0,64 | 0,04 | 0,52 | 0,13 | 10 | NC |
| Myogenin (AU/fiber no.) | CSA | 0,7 | 0,03 | 0,39 | 0,26 | 10 | NC |
| PCNA (AU/fiber no.) | CSA | -0,06 | 0,88 | 0,04 | 0,92 | 10 | NC |
| TGFß (AU/fiber no.) | CSA | 0,3 | 0,39 | 0,49 | 0,15 | 10 | NC |
| TNF-a (AU) | CSA | -0,54 | 0,11 | -0,6 | 0,07 | 10 | CIA |
| IL-1ß (AU) | CSA | 0,38 | 0,28 | 0,31 | 0,39 | 10 | CIA |
| BMP7 (AU) | CSA | -0,14 | 0,7 | -0,36 | 0,31 | 10 | CIA |
| IL-10 (AU) | CSA | 0,64 | 0,05 | 0,38 | 0,28 | 10 | CIA |
| IL-6 (AU) | CSA | 0,56 | 0,09 | 0,58 | 0,09 | 10 | CIA |
| MCP-1 (AU) | CSA | 0,46 | 0,18 | 0,28 | 0,43 | 10 | CIA |
| MIF (AU) | CSA | 0,12 | 0,75 | 0,05 | 0,89 | 10 | CIA |
| Id2 (AU) | CSA | -0,6 | 0,07 | -0,85 | <0.01 | 10 | CIA |
| Mafbx (AU) | CSA | -0,37 | 0,3 | -0,49 | 0,15 | 10 | CIA |
| NFκB (AU) | CSA | -0,39 | 0,26 | -0,33 | 0,35 | 10 | CIA |
| pSmad1/5/8 (AU) | CSA | -0,01 | 0,98 | 0,08 | 0,84 | 10 | CIA |
| MyoD (AU) | CSA | -0,55 | 0,1 | -0,75 | 0,02 | 10 | CIA |
| Myogenin (AU) | CSA | -0,57 | 0,08 | -0,77 | 0,01 | 10 | CIA |
| PCNA (AU) | CSA | -0,28 | 0,43 | -0,32 | 0,37 | 10 | CIA |
| TGFß (AU) | CSA | -0,54 | 0,11 | -0,6 | 0,07 | 10 | CIA |
| TNF-a (AU/fiber no.) | CSA | -0,58 | 0,08 | -0,68 | 0,04 | 10 | CIA |
| IL-1ß (AU/fiber no.) | CSA | 0,8 | <0.01 | 0,71 | 0,03 | 10 | CIA |
| BMP7 (AU/fiber no.) | CSA | 0,83 | <0.01 | 0,71 | 0,03 | 10 | CIA |
| IL-10 (AU/fiber no.) | CSA | 0,74 | 0,02 | 0,62 | 0,06 | 10 | CIA |
| IL-6 (AU/fiber no.) | CSA | 0,87 | <0.01 | 0,88 | <0.01 | 10 | CIA |
| MCP-1 (AU/fiber no.) | CSA | 0,74 | 0,01 | 0,5 | 0,14 | 10 | CIA |
| MIF (AU/fiber no.) | CSA | 0,56 | 0,09 | 0,3 | 0,41 | 10 | CIA |
| Id2 (AU/fiber no.) | CSA | -0,57 | 0,08 | -0,78 | 0,01 | 10 | CIA |
| Mafbx (AU/fiber no.) | CSA | 0,58 | 0,08 | 0,13 | 0,73 | 10 | CIA |
| NFκB (AU/fiber no.) | CSA | 0,23 | 0,52 | -0,25 | 0,49 | 10 | CIA |
| pSmad1/5/8 (AU/fiber no.) | CSA | 0,26 | 0,47 | 0,37 | 0,3 | 10 | CIA |
| MyoD (AU/fiber no.) | CSA | -0,5 | 0,14 | -0,45 | 0,19 | 10 | CIA |
| Myogenin (AU/fiber no.) | CSA | -0,55 | 0,1 | -0,68 | 0,04 | 10 | CIA |
| PCNA (AU/fiber no.) | CSA | 0,24 | 0,5 | 0,21 | 0,56 | 10 | CIA |
| TGFß (AU/fiber no.) | CSA | -0,47 | 0,17 | -0,47 | 0,18 | 10 | CIA |


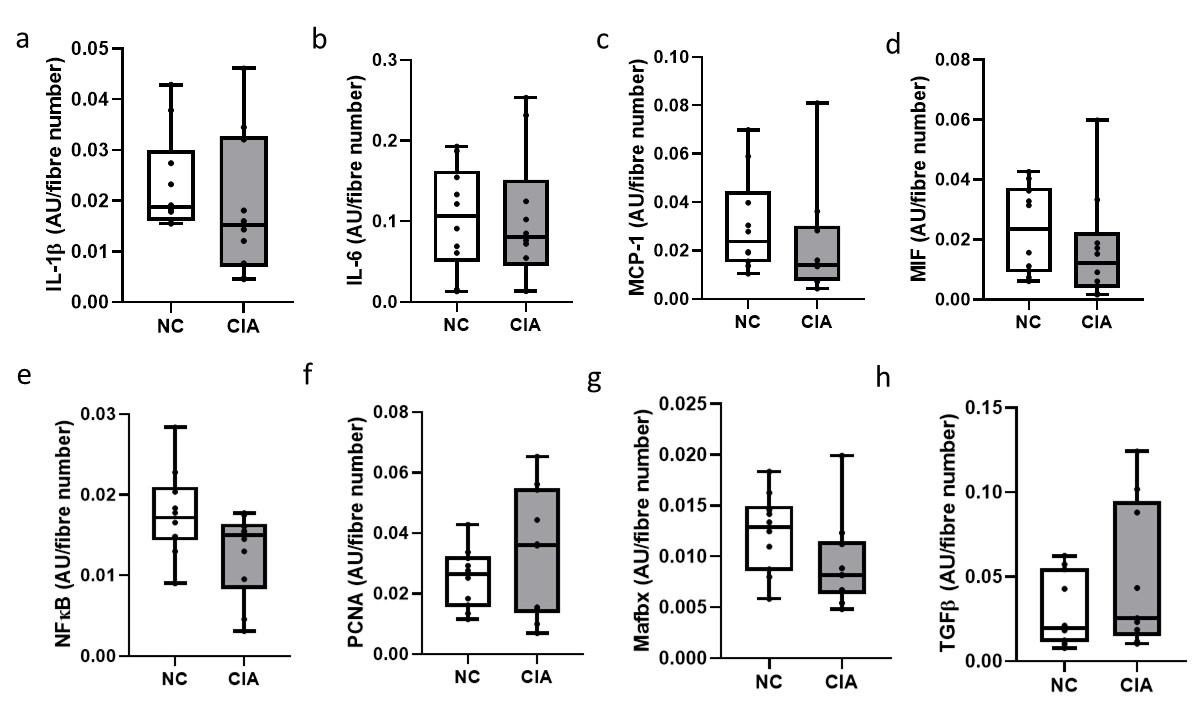
Supplementary Figure 1: Protein expression taking fiber number into consideration. a) IL-1β; b) IL-6; c) MCP-1; d) MIF; e) NFκB; f) PCNA; g) Mafbx; h) TGFβ. n=10 per group. Statistical analysis: Unpaired t-test (parametric: b, f) or Mann-Whitney test (non-parametric: a, c, d, e, g, h). Data represented as box and whisker plots indicating the highest and lowest values, the median and the interquartile range, as well as individual data points.
